# The prediction of occupational health risks of n-Hexane in small and micro enterprises within China’s printing industry using five occupational health risk assessment models

**DOI:** 10.3389/fpubh.2024.1399081

**Published:** 2024-08-21

**Authors:** Liecong Hu, Manlian Chen, Quanjin Zhong, Huipeng Chen, Xiaoxuan Cai, Muwei Cai

**Affiliations:** The Sixth People’s Hospital of Dongguan, Dongguan, Guangdong, China

**Keywords:** n-Hexane, occupational health, risk assessment, printing industry, consistency

## Abstract

**Background:**

Chronic n-Hexane poisoning is prevalent among workers in small and micro printing industries in China. Despite this, there is limited research on occupational health risk assessment in these sectors. Conducting comprehensive risk assessments at key positions and proposing effective countermeasures are essential.

**Methods:**

Data were collected from 84 key positions across 32 small and micro-sized printing enterprises. Air samples were tested for n-Hexane exposure levels in accordance with Chinese standards. Five risk assessment models were employed: COSHH, EPA, MOM, ICMM, and Technical Guide GBZ/T 289-2017 of China. The consistency of results across these models was analyzed.

**Results:**

Workers in 84 job positions were categorized into four exposure groups, with exposure to n-Hexane for 8–10 h daily, 5–6 days weekly. Most positions operated with low automation levels (96.9% in printing, 5.9% in oil blending, and 42.9% in pasting), while others were manual. Localized ventilation rates were notably low in oil blending (23.5%), cleaning (14.3%), and pasting (9.5%) groups. n-Hexane concentrations exceeded Chinese occupational limits in 15.6% of printing, 17.7% of oil blending, and 21.4% of cleaning groups. Risk assessment models identified over 60% of work groups as high risk. Significant differences (*p* < 0.05) were found among the seven risk assessment methods. Consistency analysis revealed moderate agreement between the Chinese synthesis index and exposure index methods (*k* = 0.571, *p* < 0.01).

**Conclusion:**

The Chinese synthesis and exposure index methods from Technical Guide GBZ/T 289-2017 are practical and reliable for assessing n-Hexane exposure risks in small and micro printing enterprises. Cleaning and printing roles were found to be at the highest risk for n-Hexane exposure. These findings provide valuable insights for targeted risk management strategies to protect workers’ health in the industry.

## Background

n-Hexane is a low-toxicity saturated aliphatic hydrocarbon (1). Due to its high volatility and lipophilicity, n-Hexane is widely used as a degreasing agent, cleaning solvent, and diluent in industries such as printing, electronics, and hardware manufacturing (2). Prolonged exposure to n-Hexane can result in chronic or subchronic poisoning, primarily affecting the peripheral nervous system through its metabolite, 2,5-hexanedione (3, 4).

The extensive use of n-Hexane has raised significant concerns about occupational health risks around the world. In recent years, numerous n-Hexane poisoning incidents have been reported globally, including the United States, Japan, and Italy (5–7). In China, n-Hexane has heavily utilized in various industrial sectors, particularly in small and micro printing enterprises, due to its high usage rate and volume.

Small and micro printing enterprises play a significant role in both the global and Chinese economies, contributing substantially to employment and local economic development (8). However, these businesses often face challenges in implementing effective occupational health and safety measures due to limited resources, inadequate awareness, and insufficient regulatory oversight (9). In China, small and micro enterprises constitute a significant portion of the printing industry, accounting for over 33.94% of printing businesses (10). These enterprises frequently operate in confined spaces with poor ventilation, use large quantities of organic solvents like n-Hexane, and often lack adequate personal protective equipment and safety training for workers (11). This situation places employees at an elevated risk of chemical exposure. Given these challenges, it is crucial to conduct comprehensive risk assessments to identify hazards and develop targeted control measures.

A major health concern associated with occupational exposure to n-Hexane exposure is the risk of chronic poisoning, which constitutes a significant proportion of occupational disease cases in regions such as Dongguan city, Guangdong Province, China. From 2009 to 2018, chronic n-Hexane poisoning accounted for 17.9% of total occupational disease cases in Dongguan (12). Outbreaks of chronic n-Hexane poisoning have also been reported in small and microenterprises within the electronics and printing industries, particularly in cities like Shenzhen, Guangdong Province (13). For instance, in one small company in Dongguan city, 13 workers engaged in cleaning operations developed symptoms including limb weakness, numbness, muscle atrophy, and diminished reflexes, all diagnosed as occupational chronic n-Hexane poisoning (14). These instances highlight the urgent need for effective occupational health risk assessment (OHRA) in the printing industry.

OHRA aims to identify hazardous factors in the workplace using qualitative, semi-quantitative or quantitative methods, assess their potential impact on employee health, and develop appropriate control measures (15). Various OHRA methods have been developed over the years including the U.S. Environmental Protection Agency (EPA) model (16), Singapore Ministry of Manpower (MOM) model (17), International Council on Mining and Metals (ICMM) model (18), UK Control of Substances Hazardous to Health (COSHH) model (19), and the “Technical Guidelines for Occupational Health Risk Assessment of Chemical Hazards in the Workplace” (GBZ/T 298-2017) model (20). Previous studies have often focused on assessing occupational hazard risks in specific industries or job roles, comparing different assessment methods, and proposing preventive measures (21). For instance, Chen et al. (22), Abbaslou et al. (23), and Janjani et al. (24) utilized the EPA model to evaluate job risks and hazardous factors across various industries, proposing risk reduction strategies for high-exposure roles. Pourhassan et al. (25) assessed health risks associated with metal fumes in welding processes within the metal products industry using a variety of OHRA models and found that the GBZ/T 298-2017 and COSHH models were particularly useful in predicting risks in this industry.

Traditional risk modeling approaches are often insufficient to identify enterprises that require risk control measures, prompting the development of new approaches to assess occupational health risks at the enterprise level. For instance, Huang et al. (26) proposed a hierarchical model that has proven highly effective for electronics enterprises. Xu et al. (27) developed a linear multistage model to evaluate benzene exposure across various industries. Duan et al. (28) employed a method based on ICMM, which highlighted high risks such as pneumoconiosis and noise-induced hearing loss in metal foundries. Furthermore, OHRA models have successfully reduced risks in high-risk occupations through the implementation of engineering controls. For example, Hospitals, thermometer manufacturers, and vinyl chloride producers have enhanced safety by mitigating employees’ exposure to harmful substances like benzene, formaldehyde, and mercury through risk assessment and the application of engineering controls (29–31).

Despite the availability of various OHRA models, there is a lack of studies comparing and evaluating their effectiveness in assessing n-Hexane exposure risks, particularly in China’s small and micro printing industry. This study aims to comprehensively analyze n-Hexane exposure risks within this industry by utilizing multiple OHRA models, including ICMM, COSHH, EPA, MOM, and GBZ/T 298-2017. By identifying the most effective risk assessment models and pinpointing critical at-risk positions, this research aims to develop targeted and effective risk management strategies, ultimately improving occupational health and safety in small and micro printing enterprises.

## Materials and methods

### Description of Similar Exposure Groups (SEGs)

This study focused on 84 positions across 32 small and micro-scale printing enterprises in Dongguan city, Guangdong Province, China, where production processes involve the use of n-Hexane as an organic solvent. SEGs were categorized based on varying production processes and job functions into printing, oil blending, cleaning, and pasting groups. The printing and oil blending groups primarily used ink cleaning agents, while the cleaning groups utilized agents for removing surface stains from products. The pasting groups employed agents to remove excess glue. Among the enterprises surveyed, there were 32 printing, 17 oil blending, 14 cleaning, and 21 pasting SEGs. The survey encompassed variables such as working hours, n-Hexane usage, exposure duration and concentration, ventilation conditions, process automation levels, availability of first-aid facilities, personal protective equipment (PPE), emergency protocols, and occupational health management for each group.

### Site survey and on-site testing

Following an initial profile assessment of each enterprise, an occupational health site survey was designed. This survey focused on gathering data including the number of workers, working hours, daily n-Hexane consumption, duration of exposure, implementation of engineering controls, and utilization of PPE. Post-survey, spatial distribution mapping of n-Hexane across various positions within the enterprises was conducted. Air samples for n-Hexane were collected in accordance with Chinese standards “Specifications of air sampling for hazardous substances monitoring in the workplace (GBZ 159–2004) (32)“and “Determination of toxic substances in workplace air-Part 60: Pentane, hexane, heptane, octane, and nonane (GBZ/T 300.60-2017) (33).” These samples were analyzed in a laboratory to determine the 8-h time-weighted average concentration (C-TWA) and short-term exposure concentration (C-STEL) of n-Hexane.

The determined concentrations were compared with the permissible concentration-time weighted average (PC-TWA) and short-term exposure limit (PC-STEL) specified in the Chinese standard “Occupational exposure limits for hazardous substances in the workplace-Part 1: Chemical hazards (GBZ 2.1–2019) (34).” The permissible levels of n-Hexane PC-TWA and PC-STEL were obtained based on GBZ 2.1–2019 guidelines.

### Occupational health risk assessment models

This study utilized five occupational health risk assessment models: EPA, COSHH, MOM, ICMM, and China’s GBZ/T 298-2017 standard. The EPA, COSHH, MOM, and ICMM models were selected for their widespread application in occupational hygiene globally and their proven effectiveness in various industrial settings (35). These models offer diverse approaches to assessing chemical exposures, each with unique strengths (36). China’s GBZ/T 298-2017 standard was included as it reflects national guidelines adapted from international models to suit local conditions (25). Given the limited comparative studies on these models, particularly in China’s small and micro-scale printing industries where n-Hexane is prevalent, this study aims to explore their methodological differences and assess their applicability in evaluating occupational health risks associated with n-Hexane exposure.

### ICMM model

The ICMM model employs a matrix method to evaluate risk levels. It integrates matrices linking health hazards with exposure probability in similar exposure groups or processes, and matrices linking health hazards with exposure levels and existing control measures. This comprehensive approach facilitates thorough risk identification and quantification, enabling the development of effective risk management strategies to mitigate potential health impacts (37).

### COSHH model

The COSHH model assesses chemical exposure levels and associated health risks based on occupational exposure limits (OELs) or risk phrases that categorize substances by hazard levels. It also considers physical properties such as dustiness or volatility, alongside the specific application context to evaluate exposure levels (38).

### EPA model

This study employed the EPA non-carcinogenic risk assessment model to evaluate the risks associated with n-Hexane exposure concentrations (39). The model is based on Equation 1 to determine the exposure concentration (EC) of non-carcinogenic risk and Equation 2 to calculate the hazard quotient (HQ) to determine the occupational health risk level of n-Hexane:

- Determination of the exposure concentration (EC):

(1) $$EC=\left(CA\times ET\times EF\times ED\right)/AT$$

In this equation, EC = the exposure concentration (mg/m^3^); CA = the contaminant concentration in the air (mg/m^3^); ET = the exposure time (hours/day); EF = the exposure frequency (days/year); ED = the exposure duration (year); and AT = the averaging time (ED [years] × 365 days/year×24 h/day).

- Calculate the hazard quotient (HQ):

(2) HQ=EC/RfC

In this equation, HQ is the hazard quotient, RfC is the reference exposure concentration (mg/m^3^), and the RfC of n-Hexane is 2 × 10^−3^ mg/m^3^ in this paper. A hazard quotient (HQ) value ≥1 indicates that a toxic or harmful chemical has a greater noncancer risk (unacceptable risk). If the HQ is <1, then it signifies that the toxic and harmful chemical carries a lower noncancer risk (acceptable risk) (27).

### MOM model

The MOM-developed semi-quantitative risk assessment model relies on hazard rating data, dose–response relationships, and exposure levels (40). The model comprises two methods: the exposure ratio and the exposure index methods. After comprehensive analysis, worker occupational exposure is evaluated using the risk matrix assessment Equation 3 for semi-quantitative risk assessment.


(3) $$\mathrm{Risk}=\sqrt{HR\times ER}$$

In this equation, HR = the hazard rating, and ER = the exposure level. HR can be determined based on the carcinogenicity classification established by the American Conference of Governmental Industrial Hygienists (ACGIH) and the International Agency for Research on Cancer (IARC). Alternatively, HR can be determined based on the acute toxicity of chemicals using the median lethal dose (LD50) and the median lethal concentration (LC50), which can be obtained from the Material Safety Data Sheet (MSDS). When the risk rating is not an integer, the number is rounded. Risk ratings of 1–5 represent potential risk, low risk, medium risk, high risk, and very high risk, respectively.

#### The MOM exposure ratio method

In this method, the ER is based on the ratio of the exposure level E to the OEL. The weekly time-weighted average contact (E) can be calculated using Equation 4:


(4) $$E=\left(F\times D\times M\right)/W$$

In this equation, *E* is the exposure level (mg/m^3^); *F* is the frequency of exposure per week (times per week); *D* is the average duration of each exposure (*h*); *M* is the magnitude of exposure (mg/m^3^); and *W* is the average number of working hours per week (40 h).

#### The MOM exposure index method

When air monitoring concentration results are unavailable, exposure classification can be performed according to the exposure index (EI). The ER can be calculated using Equation 5.


(5) $$ER=\sqrt[{\frac{1}{n}}]{{EI}_{1}\times {EI}_{2}\times \ldots \times {EI}_{n}}$$

In this equation, *n* = number of exposure factors, which includes vapor pressure or particle size (aerodynamic diameter), hazard control measures, weekly usage of n-Hexane, and weekly working hours. The exposure index EI is divided into 5 levels in an increasing order from 1 to 5, with 1 representing the lowest and 5 representing the highest.

### GBZ/T 298-2017 model

China’s GBZ/T 298-2017 model integrates three assessment methods: exposure ratio, exposure index, and synthesis index. Similar to the MOM model, it evaluates risk levels using ER, EI, and a comprehensive synthesis approach. The exposure ratio method calculates ER by dividing the exposure level (E) by the occupational exposure limit (OEL). The exposure index method expands on this by considering additional factors such as emergency measures, personal protective equipment, and routine chemical usage. The synthesis index method further refines risk assessment by incorporating the exposure level to OEL ratio (E/OEL), providing a comprehensive evaluation tailored to local occupational health conditions (40).

### Comparison between different assessment models

The study employed five OHRA models: EPA, COSHH, MOM, ICMM, and China’s GBZ/T 298-2017 standard. The risk results assessed by different OHRA models are not directly comparable due to variations in their assessment criteria and methodologies (41). To facilitate comparisons between the results of each model, this study introduces a risk ratio (RR) based on the literature, which represents the ratio of the risk level assessed by a certain model to the total level of its corresponding model (30, 42). In this study, the RR value represents the relative risk level of n-Hexane derived from each model, making the risk level of n-Hexane derived from different models comparable. Each model generated varied risk classifications, which were converted into five levels of risk ratios for comparison:

- Level 1 (RR = 0–0.2, potential risk)
- Level 2 (RR = 0.2–0.4, low risk)
- Level 3 (RR = 0.4–0.6, medium risk)
- Level 4 (RR = 0.6–0.8, high risk)
- Level 5 (RR = 0.8–1, very high risk)

### Statistical analysis

Statistical analysis was conducted using SPSS 24.0 software (IBM, Armonk, NY, USA). The Kruskal-Wallis H-test was employed to analyze differences in risk outcomes between the various assessment models. RR levels were assessed using Cohen’s kappa coefficient for pairwise comparisons across different risk assessment methods, categorizing risks as ‘potential,’ ‘low,’ ‘medium,’ ‘high,’ and ‘very high.’ Kappa values range from −1.00 to 1.00, with higher values indicating better agreement between results. The consistency levels are classified as follows: k < 0.20 (poor), 0.2 ≤ k < 0.4 (general), 0.4 ≤ k < 0.6 (medium), 0.6 ≤ k < 0.8 (strong), and 0.8 ≤ k (very strong). The heatmap was created using the OriginPro (version 2022).

## Results

### On-site survey and test results

Table 1 summarizes the utilization of n-Hexane, exposure durations, levels of production process automation, availability of control and first-aid facilities, emergency rescue measures, occupational health management practices, and concentrations of n-Hexane among different Similar Exposure Groups (SEGs). The site survey reveals varying levels of automation across SEGs, with higher semi-automation observed in printing and pasting groups compared to manual operations predominant in oil blending and cleaning groups. Most enterprises have implemented ventilation systems; notably, local exhaust facilities are more prevalent in printing group workstations than in other groups. However, due to cost considerations, many companies lack comprehensive emergency response facilities. Approximately half of the companies provide inadequate or improperly worn personal protective equipment (PPE) due to convenience. Emergency response measures are less comprehensive in printing and oil blending groups compared to other SEGs. More than half of the companies surveyed lack proper occupational health management protocols.

**Table 1:** Survey results of SEGs exposed to n-Hexane.

SEG		Printing groups	Oil blending groups	Cleaning groups	pasting groups
Number of groups		32	17	14	21
Number of workers per group		1–24	1–5	1–2	1–12
Duration of work (months)		63.5 (9–148)	64 (8–136)	78.5 (35–147)	106 (17–146)
Daily usage (kg/L)		3.57 (0.25–106.94)	2.25 (0.21–51.39)	0.51 (0.21–80)	1.04 (0.35–90.28)
Weekly usage (kg/L)		19.43 (1.25–641.67)	13.50 (1.25–308.33)	3.06 (1.05–480)	6.25 (2.08–541.67)
Hours of work per day		8 (8–10)	8 (8–10)	8 (8–10)	8 (8–10)
Days of work per week		6 (5–6)	6 (5–6)	6 (5–6)	6 (5–6)
C-TWA (mg/m^3^)		8.10 (0.07–91.40)	4.80 (0.07–88.00)	6.15 (0.07–143.70)	0.10 (0.07–27.60)
C-STEL (mg/m^3^)		24.70 (0.30–324.70)	11.90 (0.30–333.60)	13.60 (0.30–290.60)	0.90 (0.30–54.10)
E/OEL		0.22 (0.002–1.804)	0.082 (0.002–1.853)	0.127 (0.002–1.614)	0.005 (0.002–0.353)
Result					
	C-TWA disqualified	3.1% (1/32)	0 (0/17)	7.1% (1/14)	0 (0/21)
	C-STEL disqualified	15.6% (5/32)	17.7% (3/17)	21.4% (3/14)	0 (0/21)
Automation level					
	Full automation	0 (0/32)	0 (0/17)	0 (0/14)	0 (0/21)
	Semi-automation	96.9% (31/32)	5.9% (1/17)	0(0/14)	42.9% (9/21)
	Manual operation	3.1% (1/32)	94.1% (16/17)	100% (14/14)	57.1% (12/21)
Ventilation					
	General ventilation	50% (16/32)	76.5% (13/17)	85.7% (12/14)	90.5% (19/21)
	Local exhaust ventilation	50% (16/32)	23.5% (4/17)	14.3% (2/14)	9.5% (2/21)
First-aid facility equipped		40.6% (13/32)	35.3% (6/17)	28.6% (4/14)	38.1% (8/21)
Personal protective equipment					
	Equipped	46.9% (15/32)	41.2% (7/17)	42.9% (6/14)	38.1% (8/21)
	Used or worn	31.2% (10/32)	35.3% (6/17)	35.7% (5/14)	23.8% (5/21)
Emergency rescue measures complete		15.6% (5/32)	17.7% (3/17)	21.4% (3/14)	28.6% (6/21)
Occupational health management					
	Performs well	15.6% (5/32)	17.7% (3/17)	7.1% (1/14)	14.3% (3/21)
	Performs poorly	34.4% (11/32)	29.4% (5/17)	42.9% (6/14)	28.6% (6/21)
	Lack of management	50% (16/32)	52.9% (9/17)	50% (7/14)	57.1% (12/21)

C-STEL, short-term exposure concentration; C-TWA, 8-h time weighted average concentration; E/OEL, the ratio of exposure concentration to the occupational exposure limit; the results here represent the larger ratios of C-TWA/PC-TWA and C-STEL/PC-STEL; PC-TWA, the permissible concentration-time weighted average; PC-STEL, the permissible concentration-short term exposure limit; SEG, the similar exposure group.

The n-Hexane concentrations in the pasting group were significantly lower compared to the other groups. Specifically, in the printing groups, the average 8-h Time-Weighted Average (C-TWA) concentration of n-Hexane was 8.10 mg/m^3^ (range: 0.07–91.40 mg/m^3^), with an average Short-Term Exposure Limit (C-STEL) of 24.70 mg/m^3^ (range: 0.30–324.70 mg/m^3^). In the oil blending groups, the average C-TWA was 4.80 mg/m^3^ (range: 0.07–88.00 mg/m^3^), and the average C-STEL was 11.90 mg/m^3^ (range: 0.30–333.60 mg/m^3^). For the cleaning groups, the average C-TWA was 6.15 mg/m^3^ (range: 0.07–143.70 mg/m^3^), with an average C-STEL of 13.60 mg/m^3^ (range: 0.30–290.60 mg/m^3^). The pasting groups exhibited an average C-TWA of 0.10 mg/m^3^ (range: 0.07–27.60 mg/m^3^), with an average C-STEL of 0.90 mg/m^3^ (range: 0.30–54.10 mg/m^3^). Notably, the printing group recorded higher average C-STEL and C-TWA values compared to the other SEGs. Results indicated that 3.1% of the printing group and 7.1% of the cleaning group exceeded the Permissible Concentration-Time Weighted Average (PC-TWA) standard. Additionally, 15.6% of the printing group, 17.7% of the oil blending group, and 21.4% of the cleaning group exceeded the Permissible Concentration-Short-Term Exposure Limit (PC-STEL) standard.

### Risk assessment results

Table 2 summarizes the risk assessment findings from three models regarding prolonged n-Hexane exposure (R48) through inhalation or skin absorption, which poses significant workplace health risks. According to the ICMM model, exposure risks range from low to high, with health consequences rated at level 2 across all groups. The COSHH model rates the hazard rating (HR) of n-Hexane as D, indicating high risks (ER levels 2–3) for all exposed workers. The EPA model reveals that 60.7% of n-Hexane-exposed workgroups had a Hazard Quotient (HQ) > 1: printing (76.5%), oil blending (70.6%), cleaning (71.4%), and pasting (14.3%). This suggests a heightened risk of non-carcinogenic effects across most n-Hexane-exposed workgroups.

**Table 2:** Evaluation results of three risk assessment models of n-Hexane.

SEG	Number of groups	ICMM model		COSHH model		EPA model	
		PEO	Risk level	ER	Risk level	HR	Risk level
Printing groups	32	Low (23/32) and high (3/32)	Potential risk (71.9%) and very high risk (13.0%)	2–3	4 (Very high risk)	3.40 (0.02–32.44)	Unacceptable risk (76.5%)
Oil blending groups	17	Low (14/17) and high (3/17)	Potential risk (82.4%) and very high risk (21.4%)	2–3	4 (Very high risk)	1.80 (0.02–27.54)	Acceptable risk (29.4%)
Cleaning groups	14	Low (10/14) and high (1/10)	Potential risk (71.4%) and very high risk (10.0%)	2–3	4 (Very high risk)	2.58 (0.02–44.97)	Unacceptable risk (71.4%)
Pasting groups	21	Low (21/21)	Potential risk (100%)	2	3 (High risk)	0.04 (0.02–10.36)	Acceptable risk (85.7%)
Total	84	Low (68/84) and high (7/84)	Potential risk (81.0%) and very high risk (10.3%)	2–3	4 (Very high risk)	1.79 (0.02–44.97)	Unacceptable risk (60.7%)

ICMM, International Council on Mining and Metals; COSHH, UK Control of Substances Hazardous to Health; EPA, U.S. Environmental Protection Agency; MOM, The Singapore Ministry of Manpower. PEO, the probability of exposure occurring. ER, exposure rating; HR, hazard rating. HQ, the hazard quotient; IR, the excess personal risk of carcinogenic inhalation; SEG, the similar exposure group.

Table 3 presents the n-Hexane risk assessment results from four semi-quantitative models, categorizing HR into five levels based on acute toxicity (LD50 = 25 g/kg). The MOM exposure ratio method indicates risk levels ranging from 1 to 3, with 18.8% (printing), 17.6% (oil blending), 21.4% (cleaning), and 19.0% (pasting) of groups classified at medium risk. The MOM exposure index method categorizes all workgroups with risk levels between 2 and 3, with printing and oil blending groups at 100% medium risk, and cleaning and pasting groups predominantly at medium risk. The Chinese exposure index method similarly places groups at risk levels 2–3, with 83.3% at medium risk and 16.7% at low risk. The Chinese synthesis index method yields comparable results to the exposure index method, with medium risk prevalent among printing (68.8%), oil blending (64.7%), cleaning (64.3%), and pasting (66.7%) groups.

**Table 3:** Evaluation results of semi-quantitative risk assessment models of n-Hexane.

SEG	Number of groups	Risk level	MOM exposure ratio method	MOM exposure index method	Chinese exposure index method	Chinese synthesis index method
Printing groups	32	1	28.1% (9/32)	0	0	0
		2	53.1% (17/32)	0	15.6% (5/32)	31.3% (10/32)
		3	18.8% (6/32)	100.0% (32/32)	84.4% (27/32)	68.8% (22/32)
Oil blending groups	17	1	47.1% (8/17)	0	0	0
		2	35.3% (6/17)	0	23.5% (4/17)	35.3% (6/17)
		3	17.6% (3/17)	100.0% (17/17)	76.5% (13/17)	64.7% (11/17)
Cleaning groups	14	1	42.9% (6/14)	0	0	0
		2	35.7% (5/14)	14.3% (2/14)	21.4% (3/14)	35.7% (5/14)
		3	21.4% (3/14)	85.7% (12/14)	78.6% (11/14)	64.3% (9/14)
Pasting groups	21	1	51.0% (17/21)	0	0	0
		2	19.0% (4/21)	9.5% (2/21)	9.5% (2/21)	33.3% (7/21)
		3	0	90.5% (19/21)	90.5% (19/21)	66.7% (14/21)
Total	84	1	74.6% (40/84)	0	0	0
		2	38.1% (32/84)	4.8% (4/84)	16.7% (14/84)	33.3% (28/84)
		3	14.3% (12/84)	95.2% (80/84)	83.3% (70/84)	66.7% (56/84)

MOM, The Singapore Ministry of Manpower; SEG, the similar exposure group.

**Figure 1:**
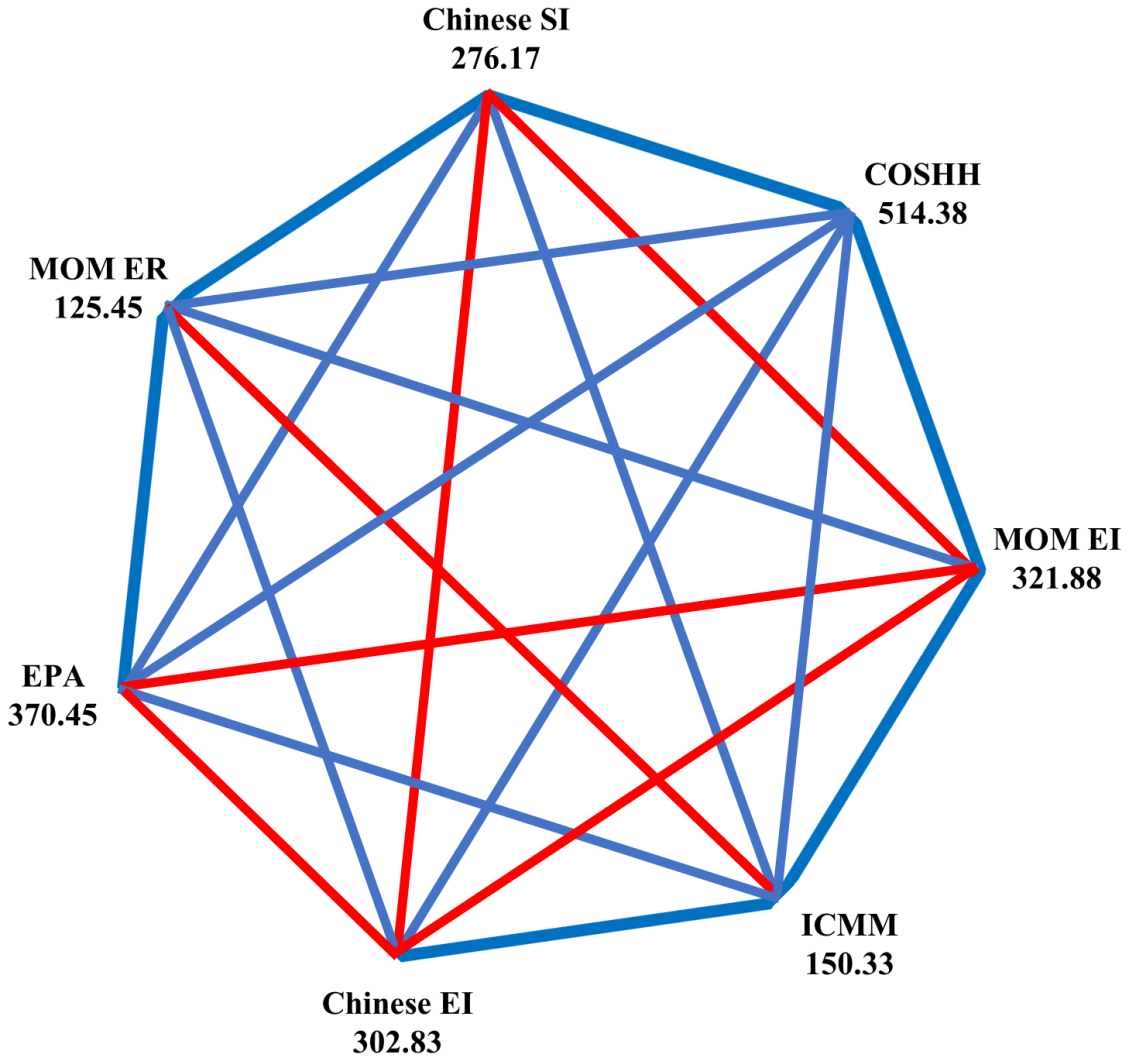
A graph of nodes representing the comparison of RR between different models. The blue color represents statistically significant differences, the red color represents no statistical differences, and the numbers represent the mean rank of the samples in each model. ICMM, ICMM model; COSHH, COSHH model; EPA, EPA model; MOM ER, MOM exposure ratio method; MOM EI, MOM exposure index method; Chinese EI, Chinese exposure index method; Chinese SI, Chinese synthesis index method.

To facilitate comparison, risk levels were converted to relative risk (RR) in Table 4. Due to variance heterogeneity among RR from different models, the Kruskal-Wallis H-test was employed, revealing significant differences across the seven assessment methods (*p* < 0.05). Pairwise comparisons indicated that the COSHH model yielded the highest risk level (median RR = 1), significantly differing from other models (*p* < 0.05). The EPA model (median RR = 0.8) showed no significant difference from the MOM exposure index method and Chinese exposure index method but differed from other methods (*p* < 0.05). The MOM exposure index method, Chinese exposure index method, and synthesis index method displayed similar risk levels (median RR = 0.6). The MOM exposure ratio method exhibited a relatively lower risk level (median RR = 0.4). The ICMM model (median RR = 0.25) showed no significant difference from the MOM exposure ratio method but differed from other methods (*p* < 0.05) (Figure 1).

**Table 4:** Risk ratio transformation for risk levels of multiple risk assessment models.

SEG	Number of groups	RR	R_a_	ICMM model	COSHH model	EPA model	MOM exposure ratio method	MOM exposure index method	Chinese exposure index method	Chinese synthesis index method
Printing groups	32	0.2–0.4	2	71.9% (23/32)	0	6.3% (2/32)	28.1% (9/32)	0	0	0
		0.4–0.6	3	9.4% (3/32)	0	6.3% (2/32)	53.1% (17/32)	0	15.6% (5/32)	31.3% (10/32)
		0.6–0.8	4	18.8% (6/32)	15.6% (5/32)	6.3% (2/32)	18.8% (6/32)	100% (32/32)	84.4% (27/32)	68.8% (22/32)
		0.8–1	5	0	84.4% (27/32)	81.3% (26/32)	0	0	0	0
Oil blending groups	17	0.2–0.4	2	82.4% (14/17)	0	11.8% (2/17)	47.1% (8/17)	0	0	0
		0.4–0.6	3	0	0	11.8% (2/17)	35.3% (6/17)	0	23.5% (4/17)	35.3% (6/17)
		0.6–0.8	4	17.6% (3/17)	29.4% (5/17)	5.9% (1/17)	17.6% (3/17)	100% (17/17)	76.5% (13/17)	64.7% (11/17)
		0.8–1	5	0	70.6% (12/17)	70.6% (12/17)	0	0	0	0
Cleaning groups	14	0.2–0.4	2	71.4% (10/14)	0	7.1% (1/14)	42.9% (6/14)	0	0	0
		0.4–0.6	3	7.1% (1/32)	0	7.1% (1/14)	35.7% (5/14)	14.3% (2/14)	21.4% (3/14)	35.7% (5/14)
		0.6–0.8	4	21.4% (3/14)	28.6% (4/14)	14.3% (2/14)	21.4% (3/14)	85.7% (12/14)	78.6% (11/14)	64.3% (9/14)
		0.8–1	5	0	71.4% (10/14)	71.4% (10/14)	0	0	0	0
Pasting groups	21	0.2–0.4	2	100.0% (21/21)	0	57.1% (12/21)	81% (17/21)	0	0	0
		0.4–0.6	3	0	0	23.8% (5/21)	19% (4/21)	9.5% (2/21)	9.5% (2/21)	33.3% (7/21)
		0.6–0.8	4	0	42.9% (9/21)	4.8% (1/21)	0	90.5% (19/21)	90.5% (19/21)	66.7% (14/21)
		0.8–1	5	0	57.1% (12/21)	14.3% (3/21)	0	0	0	0
Total	84	0.2–0.4	2	81.0% (68/84)	0	20.2% (17/84)	47.6% (40/84)	0	0	0
		0.4–0.6	3	4.8% (4/84)	0	11.9% (10/84)	38.1% (32/84)	4.8% (4/84)	16.7% (14/84)	33.3% (28/84)
		0.6–0.8	4	14.3% (12/84)	27.4% (23/84)	7.1% (6/84)	14.3% (12/84)	95.2% (80/84)	83.3% (70/84)	66.7% (56/84)
		0.8–1	5	0	72.6% (61/84)	60.7% (51/84)	0	0	0	0

RR, risk ration; R_a_, adjusted risk level; ICMM, International Council on Mining and Metals; COSHH, UK Control of Substances Hazardous to Health; EPA, U.S. Environmental Protection Agency; MOM, The Singapore Ministry of Manpower.

**Figure 2:**
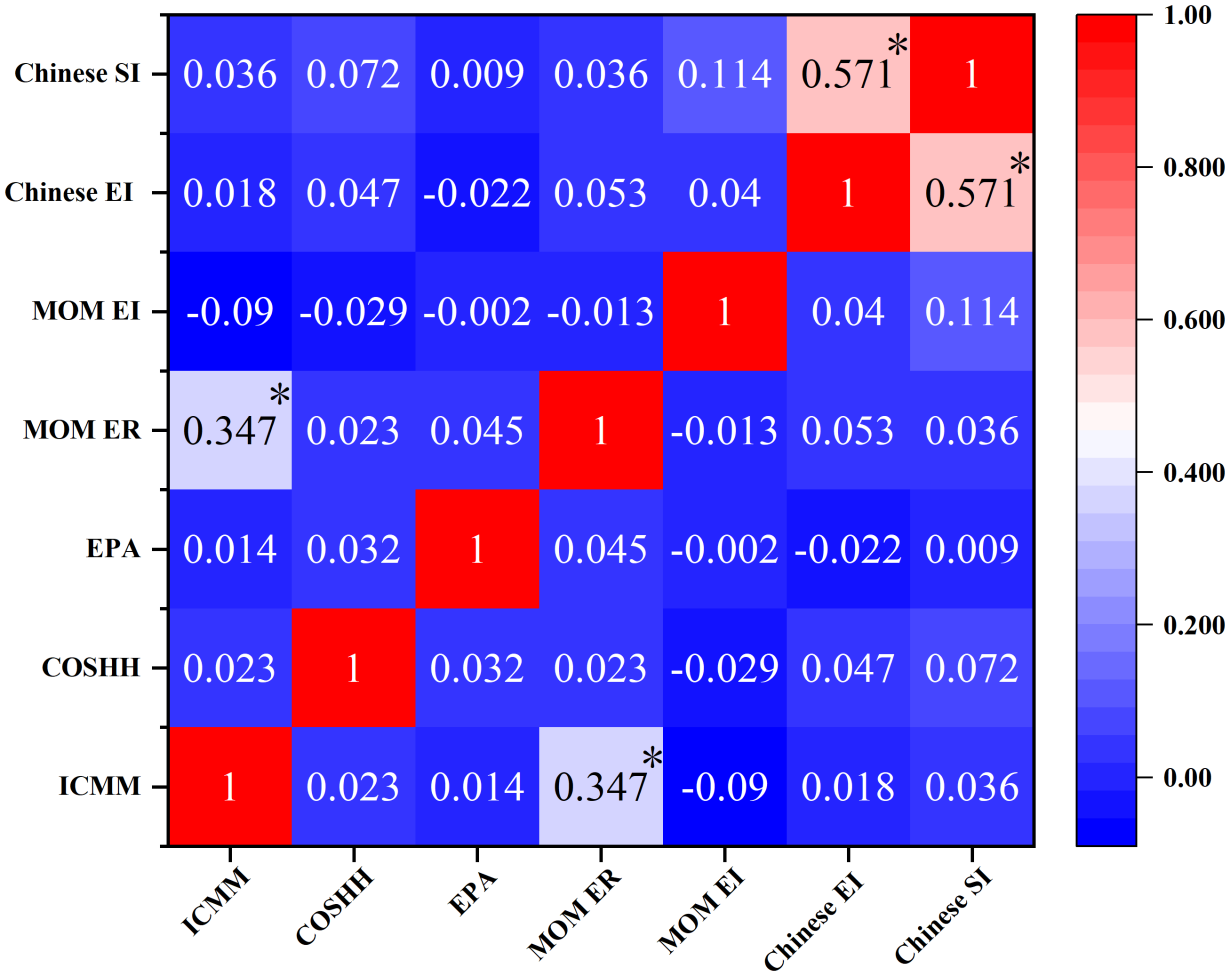
Heatmap of Cohen's Kappa results of risk assessment models of n-Hexane. ICMM, ICMM model; COSHH, COSHH model; EPA, EPA model; MOM ER, MOM exposure ratio method; MOM EI, MOM exposure index method; Chinese EI, Chinese exposure index method; Chinese SI, Chinese synthesis index method. (*p < 0.01).

Cohen’s kappa consistency tests were conducted to assess the consistency of RR levels across bidirectional ordered classification data. Figure 2 depicts the Cohen’s kappa consistency heatmap generated using OriginPro software. The MOM exposure ratio and ICMM matrix methods demonstrated general consistency (*k* = 0.347, *p* < 0.01). The Chinese synthesis and exposure index methods exhibited moderate consistency (*k* = 0.571, *p* < 0.01). Cohen’s kappa coefficients for the remaining models were below 0.2, indicating inconsistency.

## Discussion

A 2022 study revealed that out of 139 small and micro printing companies surveyed, 105 used n-Hexane-containing organic solvents (9). Workers at these companies faced the highest n-Hexane exposure risk during printing and cleaning processes (43). Our survey investigated 84 positions across 32 small and microscale printing enterprises in Dongguan city, China, a hub for economic development and concentrated printing industries. We found that 3.1% of printing and 7.1% of cleaning groups exceeded n-Hexane Permissible Concentration-Time Weighted Average (PC-TWA) limits, while 15.6% of printing, 17.7% of oil blending, and 21.4% of cleaning groups exceeded Short-Term Exposure Limit (PC-STEL) limits. These findings align with Zhu et al. (39), who reported noncompliance rates of 8.3 and 11.1% for n-Hexane PC-TWA in printing and cleaning groups, respectively.

The noncompliance in the printing group can be attributed to n-Hexane’s low boiling point (69°C) (44), extensive usage, and limited automation. Manual operations in oil blending and cleaning groups, coupled with inadequate ventilation, insufficient occupational health management, and low usage of protective equipment, likely contribute to exceedances (45). Our observations indicate that although some workshops were equipped with local exhaust ventilation, their operational efficiency often failed to meet required standards due to factors such as inadequate air change rates, infrequent maintenance, and improper system positioning relative to workstations. Zhang et al. reported that n-hexane poisoning is often caused by a lack of effective ventilation and protective facilities (46). Consequently, operators in cleaning and printing groups face heightened risks of n-Hexane poisoning.

This study utilized the ICMM, COSHH, EPA, MOM, and GBZ/T 298-2017 models to evaluate occupational health risks associated with n-Hexane exposure in small and micro-sized printing enterprises. The ICMM categorizes n-Hexane’s health effects at Level 2, with varying exposure possibilities from low to high and corresponding risk levels ranging between 1 and 3. Specifically, the ICMM model identifies high-risk (Level 3) proportions in printing (13.0%), oil blending (21.4%), cleaning (10.0%), and no risk in pasting (0%). Conversely, potential-risk (Level 1) proportions include printing (71.9%), oil blending (82.4%), cleaning (81.7%), and pasting (100%), attributed to n-Hexane levels below half of the Occupational Exposure Limits (OELs) being classified as Level 1 risks.

According to the COSHH model, the pasting group is rated at Level 3 (high risk), while the remaining groups are classified at Level 4 (very high risk). The EPA model highlights a high non-carcinogenic risk for the Printing, Oil Blending, and Cleaning Groups (76.5, 70.6, and 71.4% respectively), underscoring that despite automation, ventilation improvements, and enhanced management, n-Hexane concentrations ≥1/2 OELs maintain high risks due to its low Reference Concentration (RfC) of 2 × 10^−3^ mg/m^3^. Consequently, COSHH and EPA models may overestimate n-Hexane risks, whereas the ICMM may underestimate them, which is similar to the results of related literature studies (41).

Semi-quantitative models assign n-Hexane a Level 2 health risk, with risk levels across the four groups ranging from 1 to 3. The MOM exposure ratio method calculates risk levels based on vapor pressure, hazard control measures, usage, and work hours (47). Adding exposure concentration, the exposure index method of GBZ/T 298-2017’s Chinese exposure index and synthesis index methods expand on the MOM model by including emergency response, protective equipment use, and n-Hexane exposure duration, enhancing assessment comprehensiveness and accuracy (48). The Chinese exposure index method is preferred in absence of onsite n-Hexane testing, while the synthesis index method is optimal otherwise.

Results show risk levels from MOM exposure ratio method varying between 1 and 3, with 74.6% of workgroups at Level 1. The MOM exposure index, Chinese exposure index, and synthesis index methods assign identical risk assessments to the cleaning and pasting groups. For Printing and Oil Blending groups, MOM exposure index method indicates higher risks than Chinese methods. This study identifies Printing and Cleaning groups as facing highest n-Hexane exposure risks, underscoring the critical importance of risk levels, exposure concentrations, control measures, emergency provisions, and health management tailored to each group.

Furthermore, we introduced the Relative Risk (RR) index to compare the consistency of different assessment methods. The analysis revealed that, except for the EPA model, the RR of other methods generally increased by one level after standardization, with significant differences in risk ratios among the seven models (*p* < 0.05). Disparities in assessment results among various models primarily stem from differences in their evaluation methodologies and considered factors:

1. COSHH Model: this model typically indicates high or very high risk levels but shows poor consistency with other models. It focuses on inherent hazards and chemical usage while giving less consideration to actual workplace protective measures (25). Therefore, it is more suitable for preliminary risk assessments during project design phases.
2. EPA Model: non-carcinogenic risk assessments generally indicate high-risk levels, likely due to its sensitivity to potential health hazards (41). Despite n-Hexane exposure concentrations generally not exceeding occupational exposure limits in this study, the EPA model still indicates high-risk levels, which contrasts with regional occupational disease prevention and control outcomes. Hence, the EPA model is more applicable to occupational health risk assessments for low-level chemical exposures.
3. ICMM, MOM Exposure Ratio Method, and Chinese Synthesis Index Method: these methods consider on-site n-Hexane exposure concentrations but yield different assessment results. The ICMM and MOM methods show relatively lower RR exposure levels, while the Chinese Synthesis Index Method indicates higher levels, reflecting differences in assessment elements and methodologies among the models. The Chinese Synthesis Index Method, incorporating multiple factors, provides a more comprehensive and objective assessment.
4. MOM Exposure Index Method and Chinese Exposure Index Method: although these methods do not directly consider actual exposure levels, their assessment elements are similar. The Chinese Exposure Index Method incorporates enterprise-specific occupational health management status, thus offering higher accuracy and practical application value.
5. Chinese Synthesis Index Method and Exposure Ratio Method: these two methods demonstrate good consistency (Kappa = 0.571, *p* < 0.01). The Chinese Synthesis Index Method further incorporates workplace chemical factor concentrations based on the Exposure Ratio Method, resulting in a more comprehensive and objective assessment (49).

The study’s results align broadly with those of Shi et al. (40) on benzene exposure risk assessment in the printing industry, Zhu et al. (39) on n-Hexane exposure risk assessment in the electronics industry, and Su et al. (38) on trichloroethylene exposure risk assessment in the electroplating industry.

In conclusion, the Chinese Exposure Index and Synthesis Index Methods (GBZ/T 298-2017 model) demonstrate significant advantages in assessing risks for small and micro-sized printing enterprises. These methods provide comprehensive and objective risk assessment results, effectively guiding enterprises in selectively improving protective measures, choosing cost-effective solutions, and reducing occupational health risks.

There are several strengths of the study. We addresses a critical gap in occupational health studies by focusing on China’s small and micro printing enterprises, providing practical insights tailored to the unique challenges and limited resources faced by these businesses and their workers. By employing multiple risk assessment models (ICMM, COSHH, EPA, MOM, and GBZ/T 298-2017), we offer a comprehensive perspective on risk evaluation. This integrative approach not only elucidates the advantages and limitations of each model, helping to select appropriate assessment methodologies, but also enhances the reliability and practicality of the results. Furthermore, our reliance on on-site survey and measurement data bolsters the reliability of our findings.

However, this study has limitations. Geographical concentration may limit the generalizability of our findings to other regions of China. Moreover, our assessment primarily relies on short-term exposure data (50), which may not fully capture risks associated with long-term exposure. The study focuses solely on n-Hexane exposure without considering potential exposures to other chemicals or compound exposure scenarios (51). Future studies will include more printing companies of different geographic regions and types, combining exposure levels to multiple chemical occupational disease hazards and *per capita* health data, in order to obtain more accurate assessment results and provide more practical guidance to printing companies.

Several strategies can be implemented to mitigate the occupational health risks associated with n-Hexane exposure in small and micro printing enterprises. Initially, replacing n-Hexane with less toxic or non-toxic chemicals, such as anhydrous ethanol for cleaning agents, was considered. In cases where production needs cannot be met without n-Hexane, implementing automated and local exhaust ventilation systems should be prioritized as robust control measures. To enhance the efficiency and effectiveness of these controls, enterprises should ensure regular maintenance and compliance with operational standards. Additionally, enhancing occupational health management through training on the use of personal protective equipment (e.g., respirators, goggles, gloves) and establishing a system for regular inspection and replacement of protective equipment is crucial (52). Strengthening the management of n-Hexane handling and storage by sealing solvents promptly to prevent volatilization and ensuring timely cleaning of operational areas to prevent secondary volatilization is also recommended (53). Furthermore, ensuring accessible and regularly checked emergency rescue facilities, such as eyewash devices, is critical. Most importantly, regular monitoring and assessment of n-Hexane concentrations in the workplace and providing annual occupational health examinations for employees exposed to n-Hexane are essential measures.

## Conclusion

This study evaluates the occupational health risk of n-Hexane in China’s small and micro printing enterprises using multiple risk assessment models. The results highlight the practicality and reliability of the Chinese standard GBZ/T 298-2017, particularly the Chinese synthesis and exposure index methods, for assessing n-Hexane risk in this industry compared to the ICMM, EPA, MOM, and COSHH models. The findings underscore that most workgroups, especially those in cleaning and printing roles, face a high risk of n-Hexane exposure. These insights contribute to understanding occupational health risks in China’s small and micro printing industry and emphasize the necessity for targeted interventions and risk management strategies to safeguard workers’ health and safety.

## Data Availability

The original contributions presented in the study are included in the article/supplementary material, further inquiries can be directed to the corresponding author.
